# Cross-linked β-cyclodextrin and carboxymethyl cellulose hydrogels for controlled drug delivery of acyclovir

**DOI:** 10.1371/journal.pone.0172727

**Published:** 2017-02-28

**Authors:** Nadia Shamshad Malik, Mahmood Ahmad, Muhammad Usman Minhas

**Affiliations:** Faculty of Pharmacy and Alternative Medicine, The Islamia University of Bahawalpur, Bahawalpur, Pakistan; Brandeis University, UNITED STATES

## Abstract

To explore the potential role of polymers in the development of drug-delivery systems, this study investigated the use of β-cyclodextrin (β-CD), carboxymethyl cellulose (CMC), acrylic acid (AA) and *N’ N’*-methylenebis-acrylamide (MBA) in the synthesis of hydrogels for controlled drug delivery of acyclovir (ACV). Different proportions of β-CD, CMC, AA and MBA were blended with each other to fabricate hydrogels via free radical polymerization technique. Fourier transform infrared spectroscopy (FTIR) revealed successful grafting of components into the polymeric network. Thermal and morphological characterization confirmed the formation of thermodynamically stable hydrogels having porous structure. The pH-responsive behaviour of hydrogels has been documented by swelling dynamics and drug release behaviour in simulated gastrointestinal fluids. Drug release kinetics revealed controlled release behaviour of the antiviral drug acyclovir in developed polymeric network. Cross-linked β-cyclodextrin and carboxymethyl cellulose hydrogels can be used as promising candidates for the design and development of controlled drug-delivery systems.

## Introduction

Hydrogels are hydrophilic polymeric network, with the ability to absorb large quantities of water and having physicochemical characteristics which make them suitable for use in human tissues[1]. They provide good platform for the development of polymeric drug delivery systems. However, their ability for use in pharmaceuticals is mainly dependant on their mechanical strength, their drug loading potential and controlled drug release behaviour[2]. Despite all this, some drawbacks are still associated with hydrogels, when used as delivery system for drugs, such as decreased drug loading strength for hydrophobic drugs as well as less control over drug release mechanism[3]. To overcome all these drawbacks, cyclodextrin based hydrogels have been developed. Cyclodextrin (CDs) based hydrogels have the benefits of enhanced swelling capability and augmented encapsulation efficiency for the loaded drug[4].

Cyclodextrins are classified as cyclic oligosaccharides with both hydrophilic and hydrophobic surface, formed from the breakdown of starch by cyclodextringluconotransferase and having remarkable potential to form inclusion complexes with a large number of molecules[5]. Latest research revealed that polymerization of CDs can be used as a highly efficient technique for inducing desirable characteristics to them, such as stability towards heat, pH, shear forces, and swelling. Moreover, the significance of CDs as fundamental components of hydrogels lies in the fact that CD, when used for hydrogel synthesis, can host large number of drug molecules as carrier and can perform concurrently as enhancer for stability of these drug molecules[6]. Among various CDs derivatives, β-CD is considered to have a wide variety of applications in industry, owing to its proper inside cavity, lower cost of production and non-toxicity[7]. Moreover, β-CD has excellent binding properties due to which it can easily form complexes with a variety of biomolecules [8]and both organic and inorganic moieties, including a variety of drug molecules within their cavities[9]. Forces, like Weak intermolecular forces or chemical bonding are attributed for the binding mechanism[10]. Reactions, such as reduction, esterification, and crosslinking are undergone by the hydroxyl groups of β-CD to obtain products with desirable properties. Keeping in view, acrylic acid (AA) outstanding bioadhesion, pH-responsive and mechanical properties, it can be employed to develop hydrogels for pH responsive behaviour. Grafted AA can induce the pH responsiveness to developed network, thus producing drug delivery system that can exhibit controlled release, sustained release, and targeted release of drugs[11– 12]. Carboxymethylcellulose (CMC) is a vital water-soluble cellulose ether obtained from the reaction of chloroacetic acid with anhydroglucose units (AGUs) of cellulose. Due to its excellent physicochemical properties, biocompatibility, biodegradability, and low immunogenicity, CMC has remarkable prospective for use in the development of drug delivery system[13]. CMC, upon cross linking with suitable polymer or monomer has the tendency to absorb huge quantity of water [14] and undergo swelling to develop polymeric networks with desirable characteristics[15].

Acyclovir is an antiviral drug used for the treatment of herpes virus infections. To obtain the desired therapeutic outcomes, acyclovir in conventional drug delivery system needs to be administered multiple times in large doses both orally and intravenously, with a number of dose-related side effects. In order to minimize undesirable side effects associated with high dose and to reduce dosing frequency, a controlled release drug delivery system is needed that can achieve augmented drug loading and can provide efficient and desirable drug release profile. Therefore, development of a polymerization method and polymeric network of β-CD, CMC and AA that keep the required encapsulation capabilities of β-CD for ACV is desirable. The present study is aimed at developing new β–CD integrated CMC hydrogels by blending β-CD with CMC and AA. ACV is used as model drug. By varying the feed ratio of β-CD, CMC, AA and MBA, a series of hydrogels were synthesized using a free radical polymerization technique.

## Materials and method

### Materials

Acyclovir (ACV) was obtained from Brookes Pharmaceuticals (Pvt) Ltd. Karachi, Pakistan as donation. β-cyclodextrin (β-CD), carboxymethylcellulose (CMC) and N’, N’-methylenebis-acrylamide (MBA) were purchased from (Sigma-Aldrich, USA). Acrylic acid (AA) was obtained from Sigma-Aldrich (UK). The analytical grade initiator potassium persulfate (KPS) was obtained from Fluka.

### Synthesis of β-CD/CMC-co-Poly(AA) hydrogel beads

**Fig 1:**
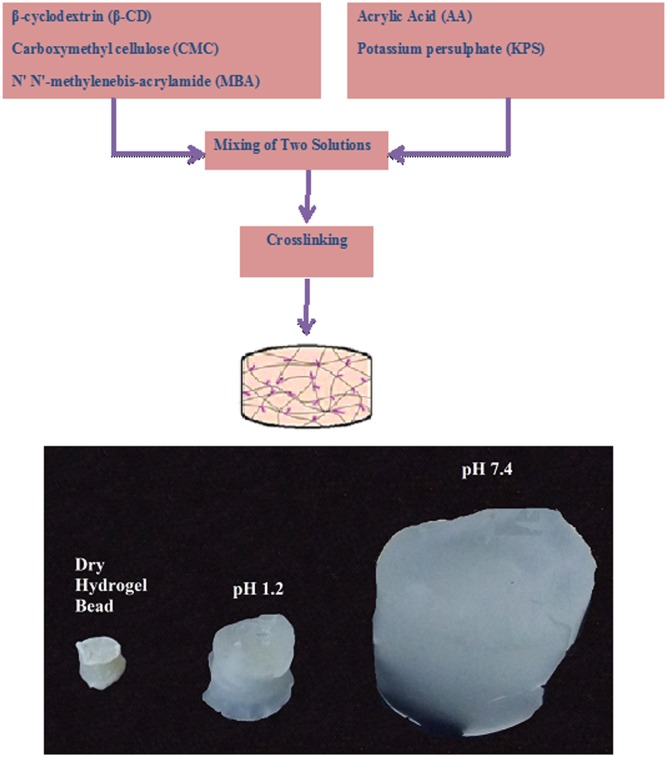
Schematic diagram for β-CD/CMC-co-Poly(AA) hydrogel synthesis.

The free radical polymerization method was used for the synthesis of β-CD/CMC-co-Poly(AA) hydrogel beads. CMC andβ-CD after weighing were added in a calculated volume of distilled water. Dissolution was carried by continuous stirring of the reaction mixture on aluminium hot plate magnetic stirrer until a homogeneous and viscous solution was obtained. Analytical grade initiator KPS was added to the above prepared polymer solution. The temperature was kept constant at 25°C, whereas stirring was maintained at 300rpm. Then monomer (AA) was weighed in a beaker on weighing balance and added into a measured amount of cross linker *N*, *N´*-methylene bis-acrylamide (MBA). Finally, monomer solution containing MBA was poured dropwise into the polymers solution under continuous stirring at 40°C until a clear solution was produced. Adjustment of final volume was done with distilled water. The reaction conditions were kept slight acidic (pH 6.5) by addition of 1.0 mL of 0.1M aqueous solution of HCl. Purging of the reaction mixture with nitrogen gas was carried out for 30 min in order to achieve uniform mixing and for removing any dissolved oxygen. Dried glass test tubes were filled with solution mixture and positioned into a water bath at 65°C for 12 h. After 12 h, test tubes were brought to room temperature for two hours. Prepared hydrogels were cut into 5mm pieces and washed with an ethanol / water mixture (70:30) to eliminate unreacted species. This process continued up to a stable value of pH was obtained. Hydrogel was further dried in a lyophilizer at -55°C until drying equilibrium. Hydrogels (FBC1-FBC9) prepared using different concentration of reactants are shown in Table 1 whereas Fig 1 shows the Schematic diagram for β-CD/CMC-co-Poly(AA) hydrogel synthesis.

**Table 1:** β-CD/CMC-co-Poly(AA) hydrogels using different concentration of reactants.

Sample code	Polymer g/100g		Monomer g/100g	Crosslinking agent g/100g	Initiator g/100g
	β-CD	CMC	AA	MBA	KPS
**FBC1**	1	12	20	0.6	0.5
**FBC2**	1	12	30	2.4	0.5
**FBC3**	1	16	30	0.6	0.5
**FBC4**	1	12	30	0.6	0.5
**FBC5**	1	8	30	0.6	0.5
**FBC6**	1	12	30	1.2	0.5
**FBC7**	8	12	30	0.6	0.5
**FBC8**	1	12	10	0.6	0.5
**FBC9**	4	12	30	0.6	0.5

### Drug loading

For drug loading, acyclovir solution (1%) was prepared in 0.2 M solution of phosphate buffer, pH 7.4. Then dried hydrogel discs were submerged in ACV solution at 25°C until achievement of constant weight. Loaded hydrogels discs were removed from the drug solution and flushed with distilled water carefully to eliminate any residual mass of drug on the exterior surface of the hydrogels discs. They were then subjected to freeze drying using the lyophilizer.

### Characterization

By employing FTIR (Tensor 27 series, Bruker Corporation, Germany), infrared spectra of the samples were obtained using attenuated total reflectance (ATR) technology accompanied by the software OPUS data collection. Over the range of 4000 cm^-1^ to 600 cm^-1^, scanning was done for grounded samples loaded on a crystal spot.

Powder X-ray diffraction (PXRD) was used to investigate the crystallinity of the hydrogels. Samples were investigated at 25°C with a diffractometer (model: X'Pert PRO, made by PAN analytical, The Netherlands). 40 kV voltage was used with a current of 28 mA. Scanning was conducted for 2h ranged 10°–45°.

JEOL analytical scanning electron microscope (JSM-6490A, Tokyo Japan)was employed to investigate structural morphology of hydrogels. Powdered samples were clamped with double adhesive tape on an aluminium stub. For coating gold on the stubs, gold sputter was utilized under an argon atmosphere.

Differential scanning calorimetry (DSC) and thermogravimetric analysis (TGA) of polymer, monomer and the hydrogel sample were carried out using a TA instrument Q2000 Series Thermal Analysis system (TA Instrument, West Sussex, UK). Samples were analyzed at a heating rate of 10°C/min under a nitrogen stream from 25°C to 500°C. Each sample was tested in triplicate.

### Swelling studies

For evaluation of pH-sensitive behaviour of prepared hydrogels, swelling experiments were conducted in simulated gastrointestinal fluid. Swelling dynamics were determined using 100 mL of 0.1-M and 0.2-M buffer solutions, pH 1.2 and 7.4, respectively maintained at 37°C. Hydrogel discs were immersed for a predetermined period in both buffer solutions for swelling. Then swollen gels were periodically taken out from the buffer solutions, weighed to determine excess weight gain and finally, returned back into their respective buffer solutions. The swelling experiments were preceded up to 72h. The swelling index was determined using Eq 1.

$$Swellingindex\;\left(Q\right)\;=\;\frac{Ms}{Md}$$ (1)

Where M_s_ indicates mass of swelling at predetermined time interval and M_d_ represents the weight of dried gel before starting the swelling experiment.

### Determination of Drug Loading Efficiency (DLE) and *In-Vitro* drug release kinetics

In order to evaluate drug loading efficiency, drug loaded hydrogel discs were crushed carefully in a mortar and pestle, weighed and then immersed in phosphate buffer solution pH 7.4, at 37°C. Stirring was continued for 24 h. Centrifugation of the drug solution was carried out at 3000 rpm to separate the supernatant layer, filtered and then assayed for ACV using UV-visible spectrophotometer at λ_max_ 256nm. DEE (%) was calculated using Eq 2.

$$\%\;Drug\;Loading\;efficiency\;=\;\frac{Actual\;loading}{Theoratical\;loading}\times \;100$$ (2)

USP dissolution apparatus II (Curio; DL-0609) was used to investigate the drug release from synthesized hydrogels. To simulate gastrointestinal conditions, drug-loaded discs were immersed in 900 mL of buffer solutions maintained at a temperature of 37°C pH 1.2 (0.1 M HCl buffer) and 7.4 (0.2 M phosphate buffer), respectively. The paddle speed was adjusted at 50 rpm. 2.5mL samples were withdrawn from the dissolution flask at specified time intervals, diluted suitably using the respective buffer solutions and were analyzed using UV–Visible spectrophotometry at λ_max_ 256nm. Drug Release Kinetics from developed hydrogels were probed employing the linear regression method using various kinetic models.

## Results and discussion

### FTIR spectroscopy

**Fig 2:**
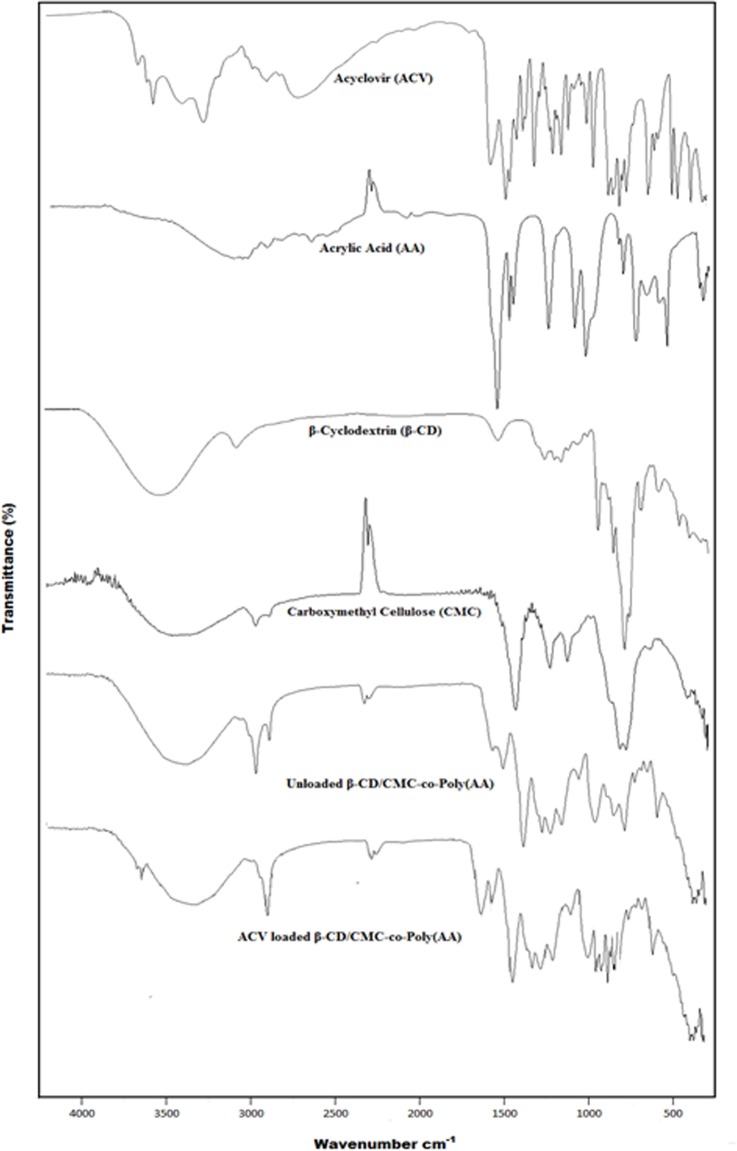
FTIR spectra of ACV, AA, β-CD, CMC, unloaded β-CD/CMC-co-Poly(AA)and ACV loaded β-CD/CMC-co-Poly(AA).

FTIR spectroscopy was used to confirm the grafting and chemical structure of the newly developed hydrogels and the individual components as shown in Fig 2. Some major peaks encountered in FTIR spectra of ACV at 3437.99 cm^-1^, 3178.44 cm^-1^, 2687.38 cm^-1^, 1707.08 cm^-1^ and 1629.67 cm^-1^ representing N–H stretching vibrations, O–H stretching vibrations, stretching movements of C—H bond, C = O stretching and N—H bending, respectively. The AA spectrum displayed prominent bands at 3016.79 cm^-1^, 1697.43 cm^-1^ and 1295.48 cm^-1^ attributed to -CH_2_ stretching vibrations, C = O stretching of carboxylic acid and C–C stretching vibrations, respectively. β-CD presented broad transmittance peak at 3303.33 cm^-1^, due to -OH stretching vibrations, asymmetric stretching vibrations of -CH were recorded at 2927.82 cm^-1^ whereas asymmetric stretching vibrations of C–O appeared at 1632.66 cm^-1^. A band at 1021.39 cm^-1^ was due to coupling vibrations of C–O and C–C. CMC showed a broad absorption band at 3352.62 cm^-1^, due to the stretching frequency of -OH group. -CH stretching vibration of -CH_2_ and -CH_3_ groups were represented by band at 2924.65 cm^-1^. The presence of a strong absorption band at 1589.69 cm^−1^ confirmed the presence of the -COO^−^ group. The bands around 1413.65cm^−1^ and 1323.89 cm^−1^ are assigned to CH_2_ scissoring and OH bending, whereas the band at 1022.19 cm^-1^ was due to the carboxymethyl ether group (CHOCH_2_-) stretching. The FTIR spectra of ACV loaded β-CD/CMC-co-Poly(AA) has demonstrated some characteristic peaks of individual components, thus indicating the formation of a new polymeric network with little or no significant modifications of functional groups of the components. Some peaks have appeared; some have shifted or disappeared in developed polymeric network. These confirm the interaction between the components at the time of hydrogel synthesis. The strong absorption bands at 1636.06cm^−1^ and 1448.48 cm^−1^ in the β-CD/CMC-co-Poly(AA)hydrogel is an indication of the presence of carbonyl groups (asymmetric -COO^−^ and symmetric -COO^−^, respectively) within the hydrogel framework. This carbonyl stretching vibration reveals that AA monomer has been grafted onto the polymeric backbone. Further, the strong characteristic absorption band of CMC at 1022.19 cm^-1^ which is due to the carboxymethyl ether group (CHOCH2-) stretching has been weakened after the reaction. This indicates that CMC participated in the grafting reaction through this group. Moreover, the new absorption bands appeared in FTIR spectra of β-CD/CMC-co-AA at 2922.53cm^−1^ and 1043.83 cm^−1^ were representing the bending vibration of C–H and bending vibration of C–C groups, respectively. These two bands, which were absent in CMC but appeared in hydrogel network, were attributed to successful crosslinking reaction between polymers β-CD, CMC and monomer AA. Another important absorption band was observed at 937.04 cm^−1^ in the spectra of the developed hydrogel was due to the vibration of α-(1 → 4) glucopyranose ring of β-CD, thus supporting that β-CD was grafted onto polymeric network.

Moreover, the FTIR spectra of ACV unloaded β-CD/CMC-co-Poly(AA) and ACV loaded β-CD/CMC-co-Poly(AA) are almost similar. However presence of some of characteristic bands of ACV with slight shift in spectra of ACV-loaded β-CD/CMC-co-AA indicates that the ACV molecules are successfully entrapped into developed hydrogel beads[16–17].

### Thermal analysis

**Fig 3:**
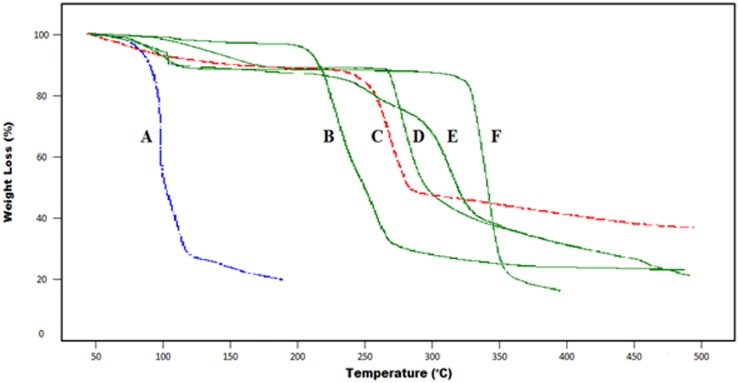
TGA curve of (A) AA, (B) unloaded β-CD/CMC-co-Poly(AA), (C) CMC, (D) ACV, (E) ACV loaded β-CD/CMC-co-Poly(AA) and (F) β-CD.

The TGA curve of all reactants and grafted hydrogel is shown in Fig 3. The initial weight loss observed for all reactants and the grafted polymeric network represents the loss of water molecules. Although, all samples were stored in air tight containers, the strongly bonded water is still present due to the high absorption capacity of reactants and grafted polymer. The heated β-CD decomposes in two steps. Despite the fact that water evaporation is responsible for the initial 13.03% weight loss, the second prominent weight loss, i.e. 18.35% was observed at 327.46°C (representing the melting range of β-CD. The main decomposition (84.28%) occurs at 359.21°C and is due to the degradation of the residual polymer. The mass of residual polymer is about 15.72% at 359.21°C. CMC exhibited two distinct decomposition phases in the thermogravimetric curve. The first phase at 55°C is associated with loss of moisture. The second degradation phase was in the range of 230–290°C and associated with decomposition of the carboxymethyl group. The weight loss observed at 290°C was 43.88%. Model drug ACV exhibited two steps decomposition phases with initial decay in the thermogram at 99.01°C, due to moisture loss. Major decay was observed at 268.69°C i.e. 93.89% representing decay of the side chain from the guanosine rings, thus leaving non-bonded guanosine, which was further degraded at higher temperatures 305.65°C. A single step decay of acrylic acid was observed at nearly 90°C. In the hydrogel formulation of ACV loaded β-CD/CMC-co-Poly(AA), the first stage of weight loss started at 100.07°C and continued up to 240.48°C. This initial 16.17% weight loss was attributed to the loss of bound water. The second larger part began at 240.48°C and continued up to 290.24°C, representing 28.04% weight loss owing to melting point temperature. The final stage of decomposition started at a temperature range of about 333.49°C, with approximately 57.97% weight loss suggesting the degradation of the more stable, probably cross-linked structures. Remarkably, the mass of residual hydrogel is about 42.03% which is quite high. The thermal profile of hydrogels with elevated residual weight represented higher thermal stability of the hydrogels than that of the individual reactants [18]. Moreover, it is evident from the TGA thermogram of both ACV loaded and unloaded hydrogel that thermal stability of developed hydrogel is increased with drug loading as shown in Fig 3. This higher thermal stability is due to the by powerful bonding between the polymers and the monomer, as a result of cross linking [19].

**Fig 4:**
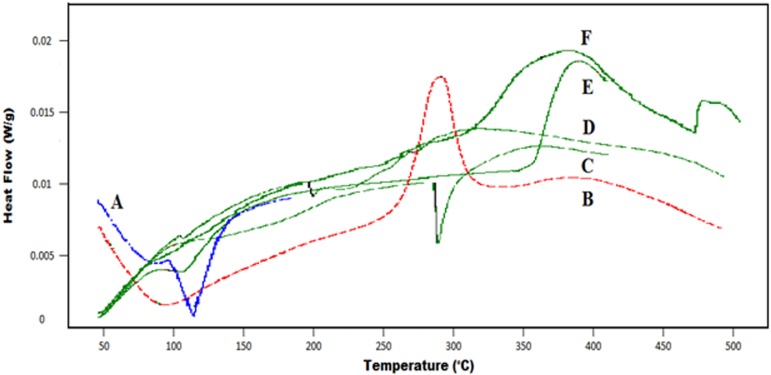
DSC Thermograms of acrylic acid, (A), CMC (B), ACV (C), unloaded β-CD/CMC-co-Poly(AA) hydrogel (D), β-CD (E) and ACV loaded β-CD/CMC-co-Poly(AA) hydrogel (F).

DSC is the thermal analysis method capable of evaluating first and second order thermal transitions, like melting(Tm), crystallization (Tc) and glass transition (Tg) phenomena. The DSC thermograms of pure β-Cyclodextrin, CMC, AA and cross-linked polymeric network are shown in the Fig 4. For chemically cross-linked ACV loaded β-CD/CMC-co-Poly(AA) hydrogel, the DSC curve exhibited a prominent and wide exothermic peak initially at 398.16°C while one endothermic peak was observed later at 462.25°C. However, in the case of the reactants, β-CD has revealed two endothermic peaks, first at 106.89°C and second at 329.46°C. CMC showed a sharp endothermic peak at 87.14°C and an exothermic peak at 279.86°C, AA revealed a sharp endothermic peak at 120°C whereas ACV displayed a major endothermic peak at 266.91°C. Peaks encountered at very early stage are relevant to loss of water whereas peaks encountered at a later stage in DSC thermogram of reactants represent either their melting point or oxidative degradation point. In the ACV loaded developed polymeric network, no endothermic peak has been observed at lower temperatures, due to the depression of melting point or glass transition temperature. Vanishing or shifting the endothermic peaks of reactants in polymeric network at earlier temperature and an appearance of a broad exothermic peak at 398.16°C and on endothermic peak at 462.25°C represents chemical modifications in the polymeric network due to higher crosslinking density [20] and branching, leading to complex formation, enhanced stability and higher Tg [21–22]. Moreover, DSC thermogram of ACV loaded hydrogel indicates that ACV no longer exists in crystalline form, possibly due to the formation of complex with the developed polymeric network. Thus, disappearance of thermal features of drug confirms the formation of complex between drug and polymeric network.

### Scanning Electron Microscopy (SEM)

**Fig 5:**
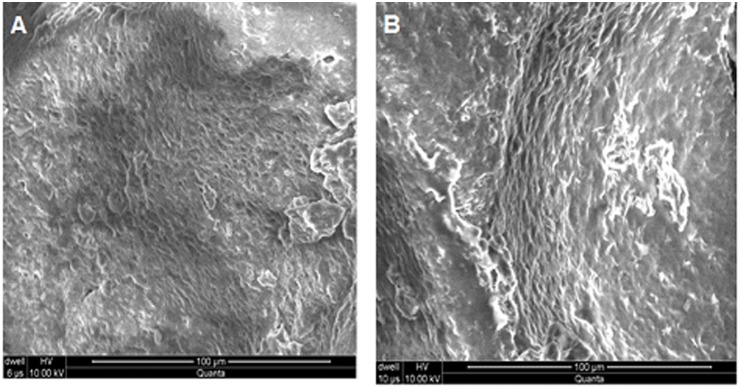
SEM images of ACV loaded β-CD/CMC-co-Poly(AA)hydrogel disc (A) at magnification of 500X (B) at magnification of 1000X.

Fig 5 shows the capillary channels in SEM images of ACV loaded β-CD/CMC-co-Poly(AA) hydrogel disc. As observed in SEM images, the structure of the hydrogel contained numerous pores interconnected to each other. This porous structure enabled water molecules to enter into the hydrogel network, thus they were considered as region of water permeation and interaction sites for biological media or buffers. Thus the porous structure is the predominant reason for the higher swelling ratios of hydrogels. Porous structure can be attributed to the presence of ionic and hydrophilic groups of the graft copolymers [23]. Moreover, the pore size of hydrogels is also interconnected with cross-linking density. The hydrogels become loose and the cross-linking density lowers and vice versa[24]. Further, difference in the drying method and freezing rate is another factor that has contributed towards the observed difference in the results of SEM analysis, as vacuum-freeze-dried hydrogels can exhibit more porous structure as seen from our results when compared with hot oven dried hydrogels.

### Powder X-Ray Diffraction (PXRD) analysis

**Fig 6:**
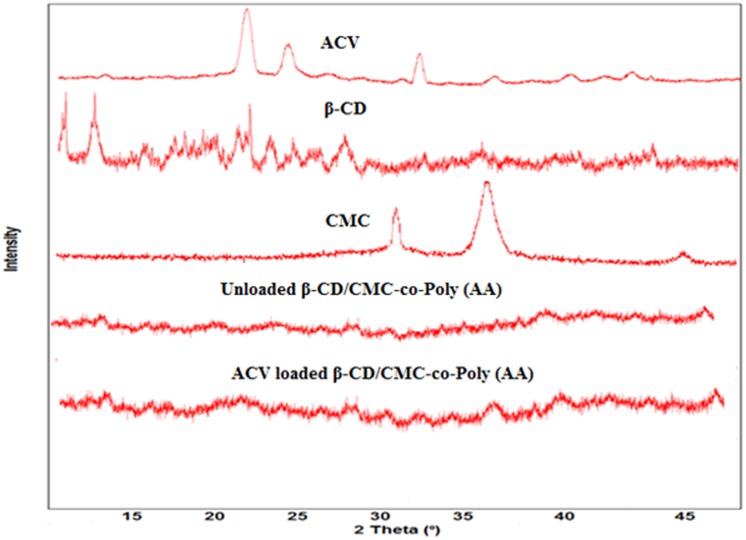
PXRD pattern of ACV, β-CD, CMC, unloaded β-CD/CMC-co-Poly(AA) and ACV loaded β-CD/CMC-co-Poly(AA).

PXRD analysis was carried out to explore the nature of reactants and grafted polymeric matrix. This nature can be either crystalline or amorphous. PXRD diffractogram of ACV, β-CD, CMC and ACV loaded β-CD/CMC-co-Poly (AA) hydrogel is shown in Fig 6. The blunt and prominent peaks in the PXRD diffractogram of ACV at 2 θ = 21.50°, 24° and 32.10° represent its crystalline state. Moreover, CMC represented two prominent peaks at 2 θ = 30.60° and 36.12°, whereas the crystalline state of β-CD was also evident in its PXRD diffractogram; displaying intense and characteristics peaks at 2 θ = 5.75°, 7.90°, 22.23°, 23.03°, 24.85° and 28°. However, in the PXRD of both unloaded and ACV loaded hydrogel beads, strong and prominent diffraction peaks disappeared. Instead, dispersed peaks were noticed, proving that interaction between reaction components is not due their mechanical mixing but rather than due to the complex formation or chemical interaction. Moreover, the crystallinity of Acyclovir decreased following its loading which is an indication of successful encapsulation of ACV within the developed polymeric network during the preparation process [25]. It further indicates that ACV was dispersed at molecular level inside developed hydrogel structure. [26].

### Swelling dynamics

**Fig 7:**
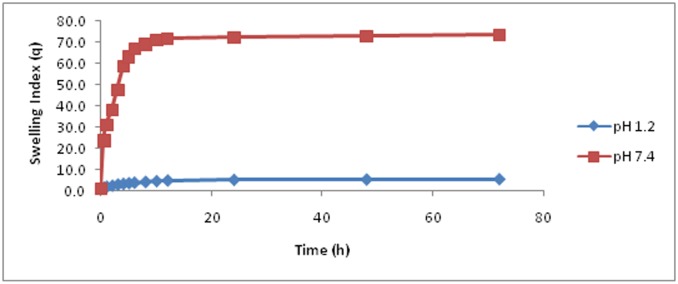
Mean swelling index for ACV loaded β-CD/CMC-co-Poly(AA) hydrogels (FBC1 toFBC9) at pH 1.2 and pH 7.4.

Swelling is one of the most essential properties of drug delivery systems to study, because it has enormous influence on drug release behaviour. Swelling is an indication of water holding ability and permeability of hydrogels. During swelling, water molecules start to diffuse into the network, leading to hydration of polar hydrophilic groups and polymer extension, until free water molecules and molecules within the network attain equilibrium. The mean of swelling index of all hydrogel formulations (FBC1-FBC9) appeared at pH 1.2 and at pH 7.4 (Fig 7). The swelling index of these synthesized hydrogels increased noticeably along with increased pH due to the presence of carboxyl groups in the polymeric network but decreased significantly at low pH, i.e. pH 1.2. This behaviour might be due to the fact that in the basic environment, the electrostatic repulsion between carboxylate anions (COO^−^) and the osmotic swelling force within hydrogel network controls expansion of the latter and ultimately swelling behaviour of hydrogels [27]. It has been found that at alkaline pH or at pH 7.4, the prevailing charged species in these hydrogels are the non-protonated carboxyl groups. Thus, due to an intra ionic repulsion between the unprotonated carboxyl groupsthese hydrogels are swollen at alkaline pH. Further, these carboxylate anions (COO^−^) in the polymeric network have a tendency of stronger solvation than non-ionic groups in aqueous media or alkaline medium. As a consequence, increased swelling of hydrogel has been observed at pH 7.4.

ACV loaded β-CD/CMC-co-Poly(AA) hydrogels did not swell at acidic pH (1.2) due to the existence of elevated proportion of protonated COOH groups, thereby lowering the ionic repulsion and strengthening the hydrogen bonding between the COOH groups, which causes hydrogels to shrink [28].

### Effect of different components of hydrogel on swelling

**Fig 8:**
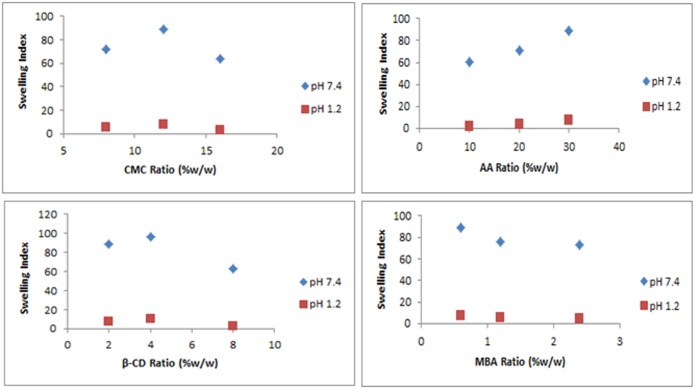
Effect of different concentration of (A) CMC, (B) AA, (C) β-CD and (D) MBA on swelling index of ACV loaded β-CD/CMC-g-Poly(AA) hydrogel.

Nine different formulations were prepared by varying the concentration of polymers (monomer and crosslinker). The effect of different concentration of reactants on the swelling behaviour of prepared hydrogels was evaluated as shown in Fig 8. Swelling of graft copolymer increased on increasing concentration of CMC up to an optimum level and keeping other reactants including β-CD, AA and MBA concentration to constant level. Above the optimum level concentration, the swelling ratio was decreased. It was observed that the swelling ratio was increased with increasing CMC concentration from 8g to 12g but decreased on further increase of CMC concentration above 12g as shown in Fig 8(A). As the CMC concentration was increased from 8g to 12g, the numbers of macromolecular radicals that can be used to graft with monomers were increased, thus leading to enhanced grafting efficiency. As a result, the swelling ratio was increased. Upon further increase from 12g to 16g polymer concentration, the viscosity of the reaction system was increased, resulting in restricted movements of macroradicals. Hence, the chain transfer reaction was also limited. All these led to a decrease of the molecular weight of the graft polymer chains and also a decrease of the swelling ratio [29]. Thus, it can be assumed from these results that optimum level concentration of CMC that can enhance swelling behaviour was up to 12g. Beyond this concentration, the swelling ratio was decreased. It has been observed that increasing the concentration of AAhydrogel formulations from 10% to 30%, the swelling ratio of ACV loaded β-CD/CMC-co-Poly(AA) hydrogels increased as shown in Fig 8(B). The carboxylic groups of acrylic acid were primarily accountable for swelling behaviour of hydrogels. It has been observed that increasing the AA concentration, the number of carboxylic group also increased within the hydrogel structure. At basic pH, the COOH groups were ionized being deprotonated to a negatively charged COO^—^group. Hydrogels having high concentration of AA possessed high concentration of negatively charged COO^—^group in their structural framework. This led to the remarkable increase in electrostatic repulsion and osmotic pressure inside the hydrogel with high AA concentration (30%) as compared to those hydrogels with less AA (10% and 20%), leading to a greater expansion of the polymeric network and enhanced the swelling ratio [30]. Fig 8(C) shows that Increasing concentration of β-CD in developed hydrogel formulations from 1% to 4% have led to an increase of the swelling index of hydrogels. This increase of the swelling index may be attributed to the hydrophilic nature of β-CD and the increased number of functional units available for grafting, thus leading to enhanced grafting efficiency. However, on further increase of concentration of β-CD, from 4% to 8% the steric effect of β-CD outweighs the ionic effect of the ionic groups of the polymer, leading to a lower swelling ratio. Steric effect of β-CD due to its bulky structure has limited its water absorbing ability and provided lesser spaces for incorporation of aqueous solution, resulting in lower swelling ratios of hydrogels having 8% β-CD in their composition [31]. Fig 8(D) shows that the swelling index of graft copolymer decreased on increasing concentration of MBA from 0.6% to 2.4%. This behaviour might be due to an increase of the cross-linking density of the developed hydrogels. An increase in crosslink density causes a decrease in flexibility of polymeric chains. High cross link density hinders the polymeric network to form hydrogen bonds with water, thus limiting their water absorbing ability and ultimately the swelling index of hydrogel [32].

### Drug Loading Efficiency (DLE) and drug release behaviour

The drug loading efficiency and the cumulative percent drug release from hydrogel formulation are shown in Table 2. It has been observed that % DLE of hydrogels demonstrated reliance on concentration of polymer porosity, the swelling behaviour of the hydrogel and extension of crosslinking. Hydrogel FBC9 exhibited maximum DLE of 92.68%, whereas FBC8 exhibited minimum DLE of 72.65%.

**Table 2:** Drug Loading Efficiency (%DLE) and percentage drug release at pH 1.2 and pH 7.4 for a period of 24 h.

Formulation code	Drug loading efficiency (%DLE)	Percentage release of acyclovir (for 24 h period)	
		pH 1.2	pH 7.4
**FBC1**	80.30	12.82	89.10
**FBC2**	85.57	18.26	91.34
**FBC3**	78.91	13.14	87.84
**FBC4**	89.54	17.30	95.19
**FBC5**	83.46	19.55	90.38
**FBC6**	87.40	19.23	94.23
**FBC7**	76.89	11.21	86.21
**FBC8**	72.65	12.17	85.57
**FBC9**	92.68	20.51	96.15

Drug loading onto these developed hydrogel beads was achieved by swelling diffusion method in which crosslinked polymeric beads were exposed to drug solution, and drug diffuses in mainly by concentration gradient. Acrylic acid being the major ionic component in the hydrogel system, so beads swells rapidly in phosphate buffer solution by virtue of their pH sensitivity and drug diffuses into the matrix quite rapidly. A major advantage of this technique is that drug loading can be achieved without using organic solvents. Mechanism by which drug was retained in β-CD/CMC complexes is not fully understood yet. As ACV may exist in multiple hydrogen bonding networks, so it may be possible that the functional group of drug have established hydrogen bonds with carboxyl functional groups of the developed polymeric network. Acyclovir can also interact with the polymeric beads due to the formation of inclusionand probably also non-inclusion complexes. Thus it can be assumed that presence of the carboxylic groups (acid groups) in the developed hydrogel beads acts as further sites for acyclovir via electrostatic interactions besides the cyclodextrin cavities.

% DLE and ACV release has been found to increase with increasing concentration of polymer(β-CD from 1% to 4% and CMC from 8% to 12%) used in reaction mixture. It could be due to increased availability of polymeric content in hydrogel, resulting in enhanced the capacity for entrapping more drug molecules into the polymeric network, thus improved drug loading and release efficiency. However on further increase in polymer ratio (β-CD from 4% to 8% and CMC from 12% to 16%), no further increase in drug loading efficiency has been observed, might be due to enhancement in the viscosity of inner phase which provides resistance for mass transfer, thereby causing a decrease in % DLE and drug release.

**Fig 9:**
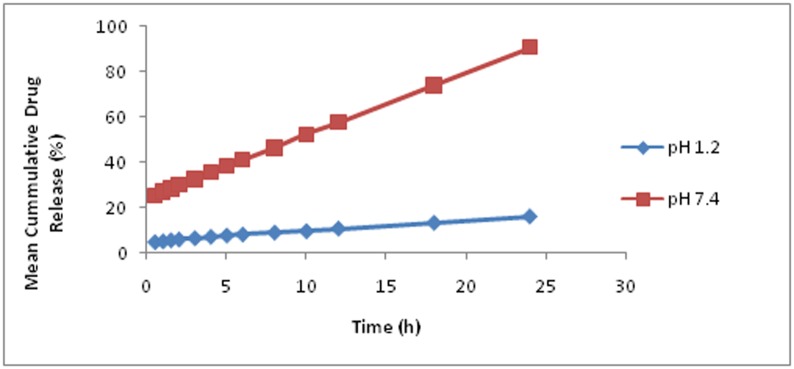
Mean cumulative drug release of ACV loaded β-CD/CMC-co-Poly(AA)hydrogels (FBC1 toFBC9) at pH 1.2 and pH 7.4.

In the acidic pH 1.2, the cumulative percentage release of acyclovir from the hydrogels was very poor, primarily due to the partial swelling of hydrogel in an acidic environment, as discussed earlier in the swelling dynamics section but increased at pH 7.4 as shown in Fig 9. Hydrogel sample FBC9 showed maximum cumulative percentage drug release (96.15%) at pH 7.4 amongst all hydrogels, which was probable due to the higher AA concentration, optimum β-CD and CMC concentration and low MBA, ensuing a more pH sensitivity and swelling profile. The cumulative percent drug release is in association with swelling dynamics as the hydrogel formulation, exhibiting highest swelling, have loaded and ultimately released maximum amount of drug. This indicates that drug release behaviour from grafted hydrogel ACV loaded β-CD/CMC-co-Poly(AA) is pH-sensitive [33].

The linear regression method was used to analyze the kinetics of acyclovir release from various hydrogel formulations as shown in Table 3. The value of regression coefficient shows very high linearity represents the best fit model, which was found to be zero order release. For all hydrogel formulations, except FBC1 and FBC9, the value of diffusion coefficient “n” is greater than 0.45, indicating non-fickian drug release [34]. However, for two samples (FBC1 and FBC9), the value of n was less than 0.45, indicating that acyclovir release kinetics in these formulations followed fickian release.

**Table 3:** Determination of regression coefficient R2, K and release exponent “n” from β-CD/CMC-co Poly(AA) hydrogels.

Sample code		Zero order kinetics		First order kinetics		Higuchi model		Korsmeyer Peppas model			Weibull model
		R^2^	K	R^2^	K	R^2^	K	R^2^	K	n	R^2^
**FBC1**	1.2	0.930	0.498	0.386	0.149	0.992	3.661	0.987	4.403	0.377	0.853
	7.4	0.998	3.022	0.328	0.268	0.955	19.223	0.973	16.631	0.598	0.970
**FBC 2**	1.2	0.994	0.694	0.548	0.151	0.963	3.822	0.975	3.058	0.651	0.945
	7.4	0.999	2.817	0.340	0.266	0.949	19.055	0.975	15.772	0.628	0.969
**FBC 3**	1.2	0.988	0.467	0.585	0.129	0.970	2.817	0.980	2.412	0.604	0.918
	7.4	0.998	2.929	0.328	0.266	0.955	18.811	0.972	16.228	0.600	0.971
**FBC 4**	1.2	0.993	0.592	0.557	0.145	0.965	3.607	0.958	2.666	0.707	0.925
	7.4	0.999	2.804	0.351	0.267	0.942	19.203	0.975	15.403	0.649	0.970
**FBC 5**	1.2	0.990	0.672	0.522	0.155	0.949	4.043	0.945	2.604	0.817	0.945
	7.4	0.999	2.724	0.334	0.267	0.956	19.218	0.980	16.644	0.596	0.961
**FBC 6**	1.2	0.997	0.632	0.513	0.156	0.955	4.119	0.976	3.326	0.647	0.957
	7.4	0.999	2.835	0.350	0.267	0.950	19.258	0.980	15.944	0.626	0.962
**FBC 7**	1.2	0.992	0.382	0.509	0.128	0.973	2.781	0.966	2.6964	0.521	0.950
	7.4	0.999	2.635	0.347	0.262	0.950	18.062	0.978	15.134	0.619	0.968
**FBC 8**	1.2	0.990	0.422	0.566	0.126	0.958	2.705	0.953	2.285	0.616	0.942
	7.4	0.999	2.675	0.354	0.260	0.953	17.639	0.979	14.233	0.645	0.964
**FBC 9**	1.2	0.990	0.623	0.355	0.178	0.979	5.528	0.980	6.282	0.413	0.952
	7.4	0.999	3.076	0.355	0.268	0.955	19.515	0.985	16.737	0.601	0.958

## Conclusions

β-CD and CMC were graft polymerized with AA, in the presence of MBA to prepare β-CD/CMC-co-Poly(AA) hydrogels and to load ACV. The FTIR results confirmed grafting copolymerization between the components. The PXRD results showed that the degree of crystallinity decreased during graft copolymerization. The SEM results indicate that the structure of hydrogel contained numerous pores interconnected to each other, thus favouring water absorption. Thermal analysis has confirmed enhanced thermal stability for the polymeric network. The presence of β-CD and CMC has conferred good mechanical properties to hydrogels with an improved drug loading and release capability for the antiviral drug acyclovir. However, pH-sensitive swelling behaviour of hydrogels was imparted mainly due to the presence of the monomer (acrylic acid). Thus, all of the above properties of the polymeric materials make them attractive and potentially appealing for the development of a controlled release drug delivery system based on hydrogels.
